# A reliable murine model of bone metastasis by injecting cancer cells through caudal arteries

**DOI:** 10.1038/s41467-018-05366-3

**Published:** 2018-07-30

**Authors:** Takahiro Kuchimaru, Naoya Kataoka, Kenji Nakagawa, Tatsuhiro Isozaki, Hitomi Miyabara, Misa Minegishi, Tetsuya Kadonosono, Shinae Kizaka-Kondoh

**Affiliations:** School of Life Science and Technology, Tokyo Institute of Technology, 4259-B60, Nagatsuta-cho, Midori-ku, Yokohama 226-8501 Japan

## Abstract

Although the current murine model of bone metastasis using intracardiac (IC) injection successfully recapitulates the process of bone metastasis, further progress in the study of bone metastasis requires a new model to circumvent some limitations of this model. Here, we present a new murine model of bone metastasis achieved by injecting cancer cells through the intra-caudal arterial (CA). This model does not require high technical proficiency, predominantly delivers cancer cells to bone marrow of hind limbs with much higher efficiency than IC injection, and greatly shortens the period of overt bone metastasis development. Moreover, CA injection barely causes acute death of mice, enabling us to inject a larger number of cancer cells to further accelerate the development of bone metastasis with a wide variety of cell lines. Our model may open a new avenue for understanding the bone metastatic processes and development of drugs preventing bone metastasis and recurrence.

## Abstract (web summary)

Reliably modelling bone metastasis in mice is essential for research. Here, the authors present a new murine model for cancer metastasis based on intra-caudal artery injection that reliably recapitulates bone metastasis.

## Introduction

Bone is one of the most common sites of metastasis for various primary tumors including prostate, breast, lung, and kidney cancers^1,2^. Although bone metastasis is associated with increased morbidity and mortality, promising therapy to prevent bone metastasis is currently unavailable. This deficiency emphasizes the need for new therapeutic approaches targeting molecular mechanisms that regulate bone metastasis and for new models to study this disease phenomenon.

Murine models of bone metastasis using intracardiac (IC) and intratibial injections have been instrumental in revealing molecular mechanisms underlying metastatic processes and translational studies for drug development^3,4^. During the past two decades, IC injection has been the gold standard to develop bone metastasis in mice^5–9^ by injecting cancer cells into the left ventricle to disseminate them to the whole body including bone marrow tissue via the arterial bloodstream, which eventually develop into metastatic colonies in the bone and other organs^10^. Unlike intratibial injection that severely damages the tibia, IC injection recapitulates the bone metastasis process, including survival of cancer cells in the bloodstream, extravasation, micro-colony formation, and metastatic progression in the intact bone marrow, and thus provides more relevant information for drug development. IC injection, however, is insufficient for rapid studies in this field, mainly owing to its requirement for high technical proficiency to exactly insert a syringe needle into the left ventricle of a mouse, causing severe cardiac stresses^3,4^. This limits the number of cancer cells that can be injected at one time, leading to limited delivery of cancer cells to the bone. Thereby analysis with IC model may bias toward cancer cell lines with relatively high metastatic ability. Furthermore, cancer cells are preferably delivered to organs other than bone, such as the lungs and liver, and often develop into lethal cancers in other organs, hampering or even terminating studies of bone metastasis with cell lines with relatively slow metastasis development. New models overcoming such limitations would accelerate basic studies and drug development for bone metastasis.

Here, we present the establishment of a new murine model that predominantly develops bone metastasis in the hind limbs at high frequency. In this model, cancer cells are injected via the caudal artery (CA) in the tail, and the technique is as easy as tail vein injection. CA injection rarely causes acute death and facilitates the injection of a large number of cancer cells, thereby greatly increasing the frequency of bone metastasis for various types of cancer cells. Therefore, CA injection provides an easy-to-use murine model to develop overt bone metastasis in a short time and could greatly facilitate studies to understand bone metastasis and to prevent them.

## Results

### CA as a new route for injection

**Fig. 1:**
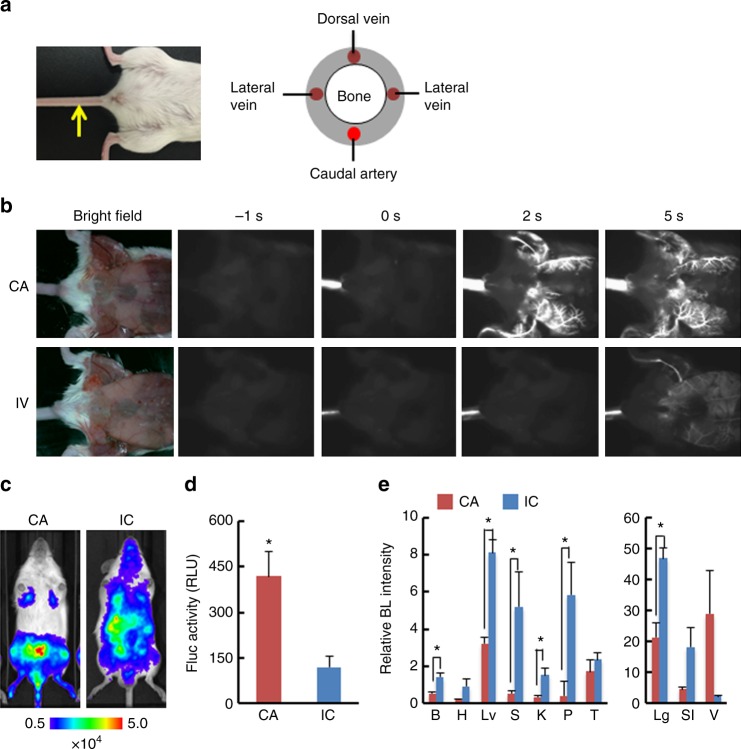
CA injection efficiently delivered cancer cells to bone marrow of a hind limb. **a** Location of caudal artery in a mouse (left) and a schematic of cross section of mouse tail (right). A yellow arrow indicates the caudal artery along the tail. **b** Comparison of fluorescence images after intra-caudal artery (CA) or tail vein (IV) injection of near-infrared II nanoparticles. **c** Representative BL images at 30 min after injecting LLC/luc cells through caudal artery (CA) or left ventricle (IC). **d** BL intensity of LLC/luc cells harvested from bone marrow of hind limbs at 30 min after CA or IC injection. *n* = 8, *P < 0.05 (two-side student’s *t*-test). Error bars indicate s.e.m. **e** Biodistribution of LLC/luc cells after CA or IC injection. Major organs were removed at 30 min after injecting LLC/luc cells and ex vivo BL imaging was performed. BL intensity of each organ was quantitatively analyzed and its relative BL intensity to the one in hind limb is shown. B brain, H heart, Lv liver, S spleen, K kidney, P pancreas, T testis, Lg lung, SI stomach and intestine, V vesicular gland. *n* = 3, *P < 0.05 (two-side student’s *t*-test)

To develop a novel murine bone metastasis model, we searched for an alternative arterial route to deliver cancer cells to bone marrow in mice. The CA was the most easily accessible route to inject cancer cells without any surgical procedures (Fig. 1a). Although cell distribution after IC injection has been well studied, no study has assessed CA-injection route. Therefore, to examine whether this route could be practically used for injection, we injected fluorescent nanoparticles emitting near-infrared II (NIR-II) fluorescence (maximum emission at 1530 nm)^11,12^. Because the nanoparticles injected via CA were thought to eventually travel to the tail vein, we compared their distributions after CA and intravenous (IV) injection by video-rate fluorescence imaging. Surprisingly, CA-injection exhibited totally different routes from IV injection: Injecting nanoparticles into the CA quickly illuminated the capillary bed in the lower body of mice, whereas nanoparticles injected via the tail vein resulted in slow and modest illumination (Fig. 1b and Supplementary Movies 1 and 2). This result implied that the CA can be a practical injection rout and may be suitable for delivery of cancer cells to the bone of hind limbs. To track the fate of cancer cells after CA injection, we used murine lung carcinoma LLC cells constitutively expressing firefly luciferase (LLC/luc). In vivo bioluminescence (BL) imaging revealed predominant delivery of LLC/luc cells to the lower body by CA injection that is technically as easy as tail vein injection (Fig. 1c and Supplementary Movie 3). CA injection delivered cancer cells three-fold more efficiently to hind-limb bone marrow than IC injection, as revealed by luciferase activity in the bone marrow 30 min after injection of LLC/luc cells (Fig. 1d). This result was well correlated to the one of ex vivo bone imaging acquired just after dissection (Supplementary Fig. 1). In addition, ex vivo BL imaging of representative organs confirmed dominant delivery of cancer cells to organs of the lower body after CA injection; in contrast, IC injection resulted in dissemination of cancer cells to various tissues (Fig. 1e and Supplementary Fig. 2). These results indicated that CA injection provide a preferable model to study bone metastasis.

### Cancer cell distribution after CA injection

**Fig. 2:**
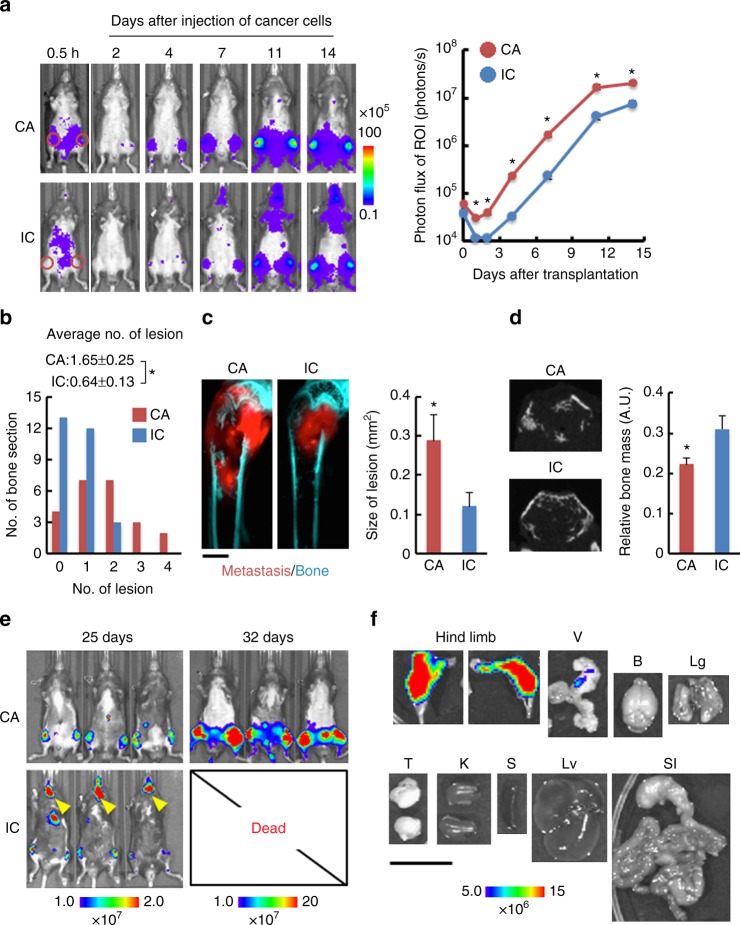
CA injection accelerated development of bone metastasis in a hind limb. **a** Representative BL images (left) and BL intensity in region of interest (ROI) (right) at indicated days after LLC/luc cells injection by CA (*n* = 16) or IC injection (*n* = 12). Red circles in the images of 0.5 h indicate ROI. *P < 0.05 (two-side student’s *t*-test). Error bars indicate s.e.m. **b** Metastatic lesions in hind limb at 7 days after CA or IC injection of LLC/luc cells. The average number of a metastatic lesion per bone section was shown above the bar graph. *n* = 23 (CA), *n* = 28 (IC), *P < 0.05 (two-side student’s *t*-test). **c** Representative fluorescence images (left) and quantitative analysis of bone metastasis in femur bones at 14 days after injecting LLC/mKO2-Rluc8.6 cells via CA or IC. Metastasis and bone were visualized with mKO2 fluorescence (red) and tissue autofluorescence (cyan), respectively. A scale bar is 500 μm. *n* = 8 (CA), *n* = 6 (IC). *P < 0.05 (two-side student’s *t*-test). **d** X-ray micro CT imaging (left) and quantitative analysis (right) of bone volume in the femur at 14 days after CA or IC injection of LLC/mKO2-Rluc8.6 cells. Representative images are transverse plane of femurs shown in **c**. *n* = 8 (CA), *n* = 6 (IC). *P < 0.05 (two-side student’s *t*-test). **e** Representative BL images of mice at 25 and 32 days after CA or IC injection of LLC/luc cells. The representative image of mice at 32 days after IC injection are not shown because all IC-injected mice died before the day. **f** Representative ex vivo BL images of organs harvested from a mouse at 32 days after CA injection of LLC/luc cells. A scale bar is 10 mm. V vesicular gland, B brain, Lg lung, T testis, K kidney, S spleen, Lv liver, SI stomach and intestine

We next compared the efficacy of bone metastasis development in mice, between CA and IC injections, by comparing luciferase activity over time after transplanting LLC/luc cells. BL intensities in the hind limbs were significantly higher after CA injection than after IC injection (Fig. 2a). Over time, growth rates were similar between these models throughout the development of bone metastasis (Fig. 2a and Supplementary Fig. 3), indicating that differences in stress between the methods, during injection and dissemination, did not affect cell proliferation in the bone marrow. Histological analysis confirmed an increase in the number of bone metastatic lesions in CA-injected mice 7 days after the injection of LLC/luc cells (Fig. 2b and Supplementary Fig. 4). In addition, significantly larger tumors were observed in the hind-limb bones of CA-injected mice at 14 days after LLC/mKO2-Rluc cell injection (Fig. 2c and Supplementary Fig. 5). X-ray micro-computed tomography (CT) imaging revealed a decrease in bone mass in CA-injected mice compared to that in IC-injected animals (Fig. 2d and Supplementary Fig. 6), confirming enhanced bone metastasis in CA-injected mice. CA injection enabled observation for more than 32 days after cancer cell injection. In contrast, IC-injected mice became weak and could not be observed after day 25 due to death (Fig. 2e). Ex vivo BL imaging of representative organs at 32 days after CA injection of LLC/luc cells revealed the development of metastasis predominantly in the hind-limb bones and some nonlethal micrometastases in vesicular glands (Fig. 2f). Most importantly, we obtained essentially similar BL imaging results in all CA-injected mice without failure.

### Improved bone metastasis development by CA injection

**Fig. 3:**
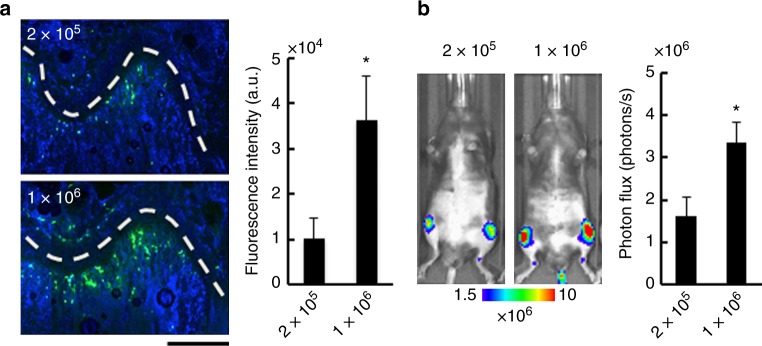
Injection of larger number of cancer cells enhances bone metastasis development. **a** Fluorescence microscopic analysis of cancer cells in bone marrow of the femur. Representative fluorescence images of the femur (left) and fluorescent intensities of cancer cells (right) at 30 min after CA injection of green-fluorescently labeled LLC/luc cells (2.0 × 10^5^ or 1.0 × 10^6^ cells). Cells were stained with Hoechst dyes (blue). White-dashed lines indicate growth plates. A scar bar is 200 μm. *n* = 4, *P < 0.05 (two-side student’s *t*-test). **b** Development of bone metastasis after injection of different number of LLC/luc cells. Representative BL images (left) and BL intensity in hind limbs (right) at 14 days after CA injection of LLC/luc cells (2.0 × 10^5^ or 1.0 × 10^6^ cells). *n* = 8, *P < 0.05 (two-side student’s *t*-test). Error bars indicate s.e.m.

Furthermore, we were able to inject a larger number of cancer cells (1 × 10^6^ cells) via CA injection without any acute death, overcoming one of the limitations of IC injection model (Supplementary Table 1). Cancer cells delivered to bone marrow in the femur increased as the number of cancer cells injected through the CA was increased (Fig. 3a). The delivery efficiency directly reflected the efficiency of bone metastasis development (Fig. 3b).

**Fig. 4:**
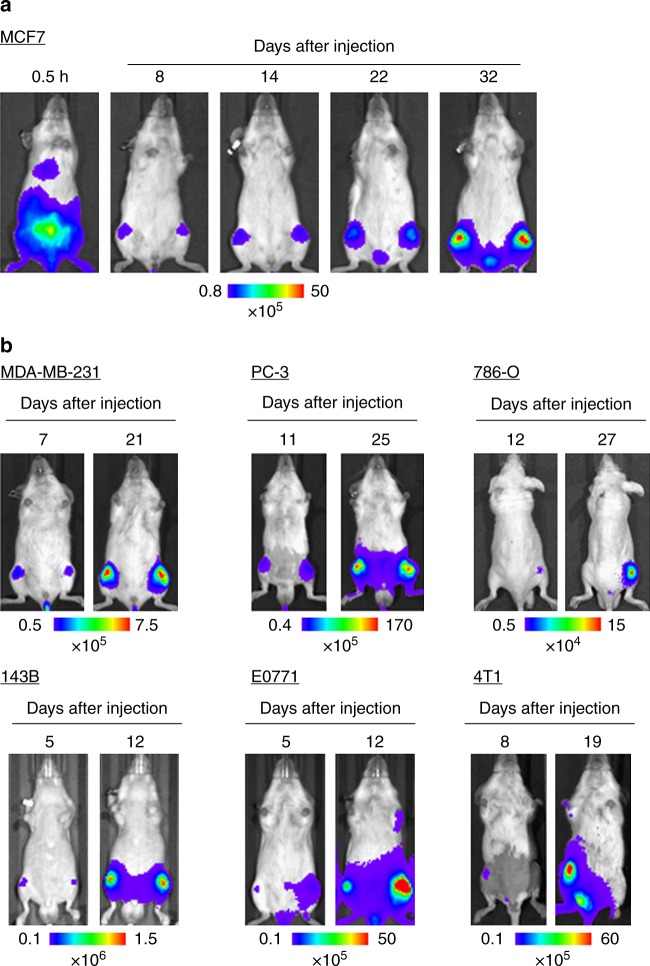
Efficient bone metastasis development with various cancer cell lines by CA injection. **a** Representative BL images at indicated days after CA injection of MCF7 constitutively expressing firefly luciferase. **b** Representative BL images at indicated days after CA injection of cancer cell lines (MDA-MB-231, PC-3, 786-O, 143B, E0771, and 4T1) constitutively express firefly luciferase

These results motivated us to examine other cancer cell lines, because cancer cell lines applicable to the IC bone metastasis model has been limited. Notably, CA-injected MCF7, which has been recognized as a non-metastatic human breast cancer cell line^13,14^, developed bone metastasis (Fig. 4a and Supplementary Fig. 7a). We further applied CA injection of several human cancer cell lines including breast (MDA-MB-231), prostate (PC-3), and kidney (786-O) cancers as well as osteosarcoma (143B). These cancers often metastasize to the bone in patients. In addition, syngeneic mouse models were examined using three cell lines including breast carcinoma E0771 that is first described here in a bone metastasis model. In vivo BL imaging confirmed that these cell lines developed bone metastasis after CA injection (Fig. 4b and Supplementary Fig. 7b). It is noteworthy that we could detect bone metastasis by 5–12 days after CA injection of all the cell line examined. Overall, the results demonstrated that CA injection provides a reliable method to develop bone metastasis by increasing the delivery efficiency of a wide variety of cancer cell lines to the bone marrow of the hind limbs in mice.

## Discussion

Here, we present a new method to establish a murine model of bone metastasis by caudal artery injection of cancer cells. The reliability (almost no failures) and easiness of this method minimize animal use and distress. The reliability is the result of the following features. (1) Because the caudal artery is visible on the body surface, injection into the caudal artery is as easy as tail vein injection. (2) Failure of CA injection is immediately detectable by low resistance upon pushing the plunger or leakage at the needle tip, and reinjection is possible at different points of the caudal artery closer to the body. (3) Successful injection can be confirmed by BL in the lower body of the mouse immediately after CA injection (Fig. 1c).

Although we first thought that the cells injected into the CA would eventually go to the tail vein, they were delivered to the bone marrow of hind limbs via a route very different from IV injection (Fig. 1b). In the CA injection model, injected cancer cells were forced to move upstream against the flow of the CA by strongly pushing them in a short time (0.1 mL per 3 s), allowing them to enter the femoral artery through the common iliac artery (see Supplementary Fig. 8)^15^. Then they rarely move further upstream to reach the interior mesenteric artery because a high BL signal was not detected in the liver, stomach, or spleen after CA injection (Fig. 1e and Supplementary Fig. 2). During these processes, cancer cells might be exposed to severe shear stresses. The in vivo growth rates, however, were similar between CA-injected and IC-injected cells (Fig. 2a and Supplementary Fig. 3). These data suggest that the cellular stress by CA-injection was, if any, similar to the one by IC injection and that the injection methods did not influence the growth of cancer cell after their delivery to the bone marrow. In addition, CA injection barely caused acute death of mice even at injection of a large number of cancer cells, allowing more cancer cells to be delivered to the bone marrow (Supplementary Table 1 and Fig. 3a). Increased delivery efficiency by CA injection increases the chance of successful colonization of cancer cells. For example, MCF7, which has been recognized as a non-metastatic cell line^13,14^, successfully developed bone metastasis as soon as 8 days after CA-injection, suggesting that the non-metastatic phenotype of these cells might be due to low homing efficiency. Further analysis will reveals the effect of hormone pre-treatment, which makes MCF7 metastatic^16^, on bone metastasis. This result suggests that our model might open up new avenues for exploring key events in bone metastasis by enabling analyses that were previously impossible using current models.

The fact that CA-injection delivers cancer cells to the organs downstream of the common iliac artery (Supplementary Fig. 8) limits the investigation for systemic effect of injected cancer cells or the development of metastasis in the bones located upper sites of the body such as skull. In contrast, this model enables the investigation of bone metastasis for a much longer time than that with the IC-injection model because of reduced incidence of lethal metastasis in other organs (Fig. 2f). This represents a great advantage for investigating cancer cell dormancy, which are key issues for bone metastasis.

Recently, intra-illiac artery (IIA) injection was described to selectively deliver cancer cells to bone marrow of a hind limb in mice, allowing efficient immunohistological analysis of metastatic colonies at the early stage of bone metastasis^17^. Although delivery efficiency to the bone might be higher by IIA injection than CA injection, the requirement of a surgical procedure and microscopic observation of IIA injection is not suitable for experiments with large numbers of mice^18^. In addition, inflammation at the site of surgery may influence the analysis of bone metastasis.

CA injection might facilitate a particular scope of studies to understand bone metastatic processes. For example, interactions between cancer cells and platelets might be a key event in bone metastasis and remain to be fully understood^19,20^. A previous study raised the issue that IC injection often causes death of transgenic mice with platelet dysfunction, probably due to lasting bleeding from the left ventricle^21^. The CA injection model may solve this problem by direct hemostasis at the injection site, enabling investigation of bone metastasis in mice with platelet dysfunction or depletion.

In the CA injection model using LLC/luc cells, metastatic formation was often detected in vesicular glands, probably because of the efficient delivery of cancer cells (Figs. 1e, 2f), but this metastasis was not lethal. Although further studies will need to reveal organotropism of various cell lines injected via the CA, our new model could represent a powerful tool for the development of drugs that target bone metastasis, in addition to facilitating research on the molecular mechanisms regulating the initiation and progression of bone metastasis.

## Methods

### Cell lines

The murine lung carcinoma cell LLC, human breast cancer cell MDA-MB-231 and MCF7, human prostate cancer cell PC-3, human renal cell adenocarcinoma 786-O, human osteosarcoma 143B and murine breast cancer cell 4T1 were obtained from ATCC (Rockville, MD, USA). Murine breast cancer cell E0771 was purchased from C3H BioSystems (Buffalo, NY, USA). Isolation of LLC/luc and MDA-MB-231/luc were described previously^9,22^. Similarly, PC-3/luc, 786-O/luc, 143B/luc, and 4T1/luc were isolated after transfection with plasmid pEF/luc by calcium phosphate method^23^. E0771 and MCF7 were stably transduced with luc2 fused with monomer KusabiraOrange2 (mKO2) using *Sleeping Beauty* transposon system^24^. To utilize the system, first pT2/CMV-MSC-SVNeo. was constructed by amplifying a fragment of CMV promoter-multi cloning site (MSC)-poly A using pcDNA3.1 plasmids (Invitrogen, Carlsbad, CA, USA) as a template and inserting the fragemt into Addgene plasmid #26553. Then pcDNA/mKO2-luc2 was constructed by inserting a luc2-mKO2 fused cDNA fragment amplified from pGL4.32 (Promega, Madison, WI, USA) and Addgene plasmid #67661 as templates, respectively and inserting the fragment into pT2/CMV-SMC-SVNeo using In-Fusion HD Cloning Kit (Clontech, Palo Alt, CA, USA). E0771 and MFC7 were co-transfected with pT2/CMV-mKO2-luc2-SVNeo and pCMV(CAT)T7-SB100 (Addgene plasmid #34879) using NEPA21 electroporator (NEPA gene, Chiba, Japan). The fluorescence/bioluminescence dual reporter-introduced cells E0771/mKO2-luc2 and MCF7/mKO2-lu2 were established from a single colony after antibiotic selection. LLC/mKO2-Rluc8.6 was established using the *Sleeping Beauty* transposon system. To construct pT2/mKO2-Rluc8.6, Rluc8.6 cDNA was amplified from pGEX/PTD-ODD-Rluc8.6 as described previously^25^. LLC/luc, LLC/mKO2-Rluc8.6, MDA-MB-231/luc, 786-O/luc, and 143B/luc were maintained at 37 °C in 5% FCS-DMEM (Nacalai Tesque, Kyoto, Japan) supplemented with penicillin (100 U/mL) and streptomycin (100 μg/mL). PC-3/luc, E0771/mKO2-luc2, and 4T1/luc were maintained at 37 °C in 10% FCS-RPMI (Nacalai Tesque, Kyoto, Japan) supplemented with penicillin (100 U/mL) and streptomycin (100 μg/mL). The cells were regularly checked for mycoplasma contamination by a mycoplasma check kit (Lonza, Basel, Switzerland) and were independently stored and recovered from the original stock every time for each experiment.

### Mice

C57B/6 mice (male), C57B/6 albino mice (male), NOD-SCID mice (female), SCID mice (male and female), BALB/c mice (female), and BALB/c-nu nude mice (male) were obtained from Charles River Laboratory Japan (Yokohama, Japan). All mice used were provided access to food and water ad libitum, and were housed in the animal facilities at Tokyo Institute of Technology. All the experimental procedures using mice were approved by the Animal Experiment Committees of Tokyo Institute of Technology (authorization number 2010006–3 and 2014005) and carried out in accordance with relevant national and international guidelines.

### NIR-II fluorescence imaging

NIR-II fluorescence images were acquired with SAI-1000 (SHIMADZU, Kyoto, Japan). Mice were injected with 50 μL OTN ceramic probe Y (Katayama Chemical Industries, Osaka, Japan) via cannulate line connected to caudal artery or tail vein, respectively. NIR-II fluorescence images were obtained using following settings: excitation/emission wavelength = 980 nm/1530 nm, laser power = 10.5 mW, and camera exposure time = 500 ms.

### In vitro analysis of cancer cells from the bone marrow

Hind-limb bones were harvested and crushed in a mortar with PBS. The bone marrow extract was centrifuged to obtain the cell pellet and the pellet was suspended into 100 μL of Passive Lysis Buffer (Promega). Fifty μL of cell lysate was mixed with an equal volume of Luciferase Assay Reagent (Promega) in a 96-well plate and then BL imaging was performed using IVIS Spectrum (PerkinElmer, Boston, MA, USA). The BL images were analyzed by Living Image 4.3 software (PerkinElmer) specialized for IVIS.

### Bone metastasis models

For CA injection, LLC/luc (2 × 10^5^ or 1 × 10^6^ cells to male C57B/6), MDA-MB-231/luc (5 × 10^5^ cells to female NOD-SCID), PC-3/luc (1 × 10^6^ cells to male SCID), 786-O/luc (1 × 10^6^ cells to male BALB/c-nu), 143B/luc (5 × 10^5^ cells to male BALB/c-nu), E0771/mKO-luc2 (2 × 10^5^ cells to female C57B/6 albino), 4T1/luc (1 × 10^3^ cells to female BALB/c), or MCF7 (1.5 × 10^6^ cells to female SCID) suspended in 100 μL PBS was injected into the caudal artery of anesthetized mice using 29 G syringe needle in a short time (<3 s). IC injection was performed as described previously^4,5^. LLC/luc (2 × 10^5^ cells) suspended in 100 μL PBS and was injected into left cardiac ventricle of 5-week-old-male C57B/6 mice. IC injection is well established and CA injection is technically as easy as IV injection. Therefore, >8 samples are adequate sample size for evaluation of metastasis growth in each experiment. Randomization and blind tests were not performed during the experiments.

### In vivo bioluminescence imaging

BL images of tumor bearing mice were acquired with IVIS Spectrum at 15 min after intraperitoneal injection of d-luciferin (50 mg/kg). The following conditions were used for image acquisition: open emission filter, exposure time = 60 s, binning = medium: 8, field of view = 12.9 × 12.9 cm, and f/stop = 1. The BL images were analyzed by Living Image 4.3 software (PerkinElmer) specialized for IVIS.

### Ex vivo bioluminescence imaging

A mouse was scarified immediately after in vivo BL imaging and major organs were removed. BL images of the organs were obtained with following conditions: open emission filter, exposure time = 30 s, binning = medium: 8, field of view = 12.9 × 12.9 cm, and f/stop = 1. The BL images were analyzed by Living Image 4.3 software (PerkinElmer) specialized for IVIS.

### Histological analysis

The isolated bone of a hind limb was fixed in 70% ethanol for 48 h, decalcified in 10% EDTA for 2 weeks, processed, and embedded in paraffin. Sectioned bones (10 μm thickness) were then stained with hematoxylin–eosin. To detect bone metastasis of mKO2-expressing cancer cells, isolated bones were embedded in OCT compound (Leica Microsystems, Wetzlar, Germany), and cryosections of the embedded bones were prepared by using CM3050 Cryostat (Leica BIOSYSTEMS), stained with Hoechst and observed under a confocal fluorescence microscope (LSM700, Carl Zeiss, Oberkochen, Germany). To quantitatively evaluate the formation of bone metastatic lesions, multiple sections were prepared from eight bones dissected from CA and IC-injected mice and metastatic lesions in each section were counted. The size of bone metastasis was quantifies by measuring area with mKO2 fluorescence with ImageJ software.

### X-ray micro CT imaging

X-ray micro CT imaging was performed using CosmoScan GX II system (Rigalu Corp., Tokyo, Japan). Dissected hind-limb bones were imaged using following parameters: 90 KV of X-ray tube voltage, 88 μA of X-ray tube current. For quantitative analysis of bone mass, X-ray CT images of transverse plane at 0.7 mm from the growth plate were analyzed using ImageJ softeware.

### Imaging of cancer cells in the bone marrow

Femur bones were harvested from mice at 30 min after CA injection of LLC/luc cells labeled with CellTracker Green CMFDA Dye (Thermo Fisher Scientific, San Jose, CA, USA). Also, mice were injected with Hoechst 33342 dyes (50 mg/kg) (Wako, Tokyo, Japan) at 5 min before isolation of bones. The isolated bone was then embedded into OCT compound to freeze it at –80 °C. The bone marrow of a femur bone was exposed using CM3050 Cryostat (Leica BIOSYSTEMS, Wetzlar, Germany) and mounted to confocal microscope (Carl Zeiss, Oberkochen, Germany) for fluorescence imaging of cancer cells in the bone marrow. Total intensity of green fluorescence in 0.5 mm from the growth plate was quantified using ImageJ 1.47.

### Statistical analysis

Data sets with similar variance were statistically analyzed. Data are presented as means ± standard error of the mean (s.e.m.) and were statistically analyzed with a two-side student’s *t*-test. *P* values of <0.05 were considered statistically significant.

### Data availability

The data that support the findings of this study are available from the corresponding author upon request.

## Electronic supplementary material

Supplementary Information

Description of Additional Supplementary Files

Supplementary Movie 1

Supplementary Movie 2

Supplementary Movie 3
