# Supplementary material for: A reliable murine model of bone metastasis by injecting cancer cells through caudal arteries
Source: Nat Commun. 2018 Jul 30;9:2981. doi: 10.1038/s41467-018-05366-3 (PMC6065368; doi:10.1038/s41467-018-05366-3)
Supplement: Supplementary file 1 — Supplementary Information [file 41467_2018_5366_MOESM1_ESM.pdf]

Supporting information

A reliable model of bone metastasis by injecting cancer cells through caudal arteries

Kuchimaru et al.

Supplementary Table 1

Incident rate of acute death after injecting cancer cells in CA and IC models.

	2×10^5 cells	1×10^6 cells
IC	0/6	7/7
CA	0/8	0/7

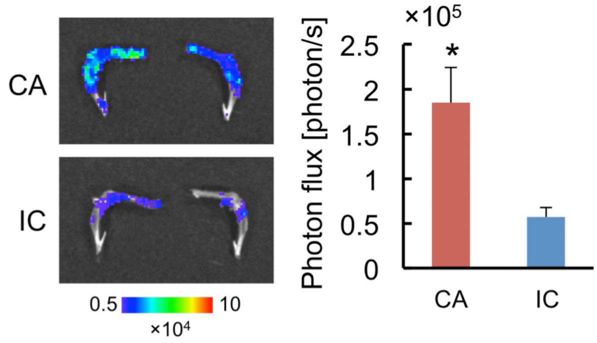

Supplementary Figure 1

Homing of cancer cells to the bone marrow. Representative *ex vivo* BL images of hind limb bones (left) at 30 min after CA or IC injection are shown. BL intensities from hind limb bones (right) were quantitatively analyzed. n=8, *P<0.05.

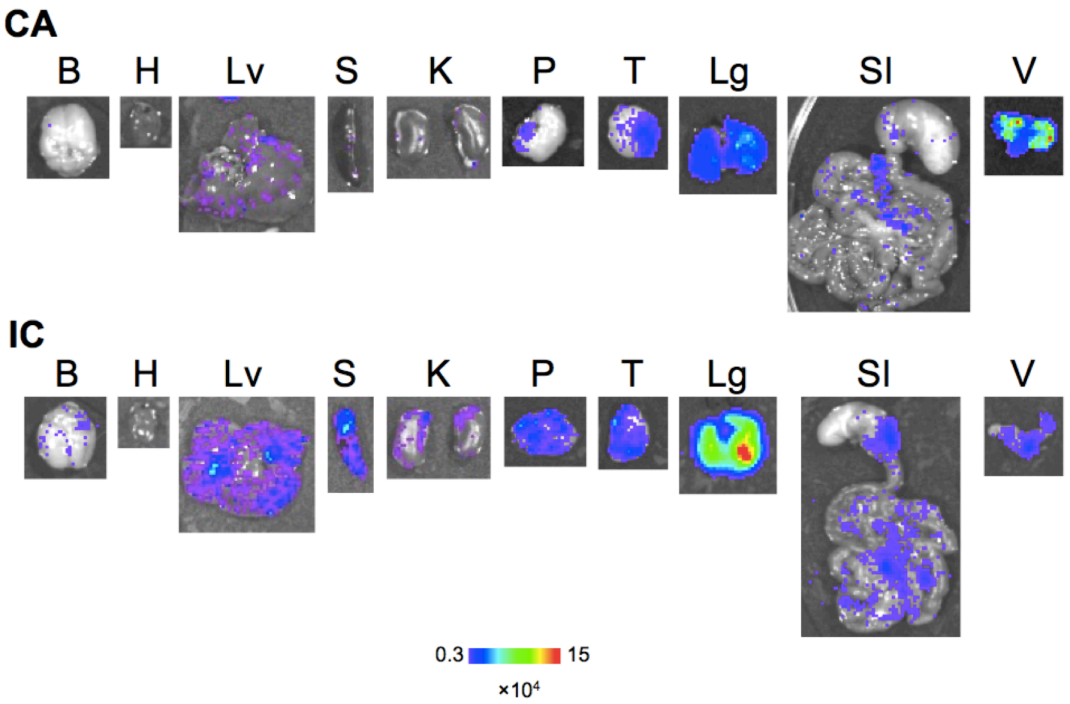

Supplementary Figure 2

***Ex vivo* images of organs shortly after cancer cell injection.** Representative *ex vivo* BL images of major organs at 30 min after CA or IC injection are shown. B: brain, H: heart, Lv: liver, S: spleen, K: kidney, P: pancreas, T: testis, Lg: lung, SI: stomach and intestine, V: vesicular gland.

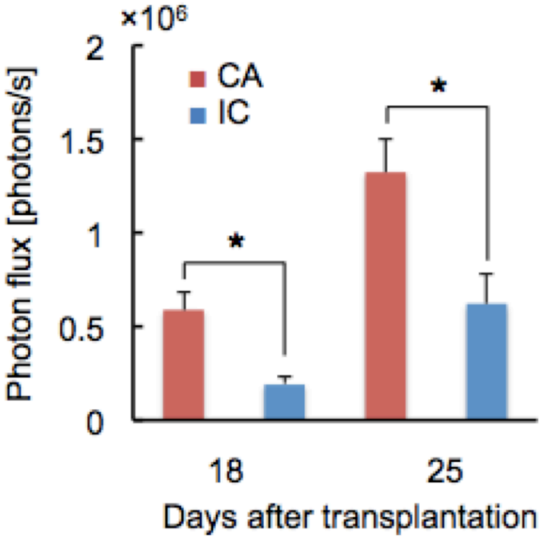

Supplementary Figure 3

Bioluminescence signal from hind limbs. BL intensities from hind limbs were compared at 18 and 25 days after injection of LLC/luc. CA (n=12), IC (n=8) for day 18. CA (n=10), IC (n=8) for day 25. *P<0.05.

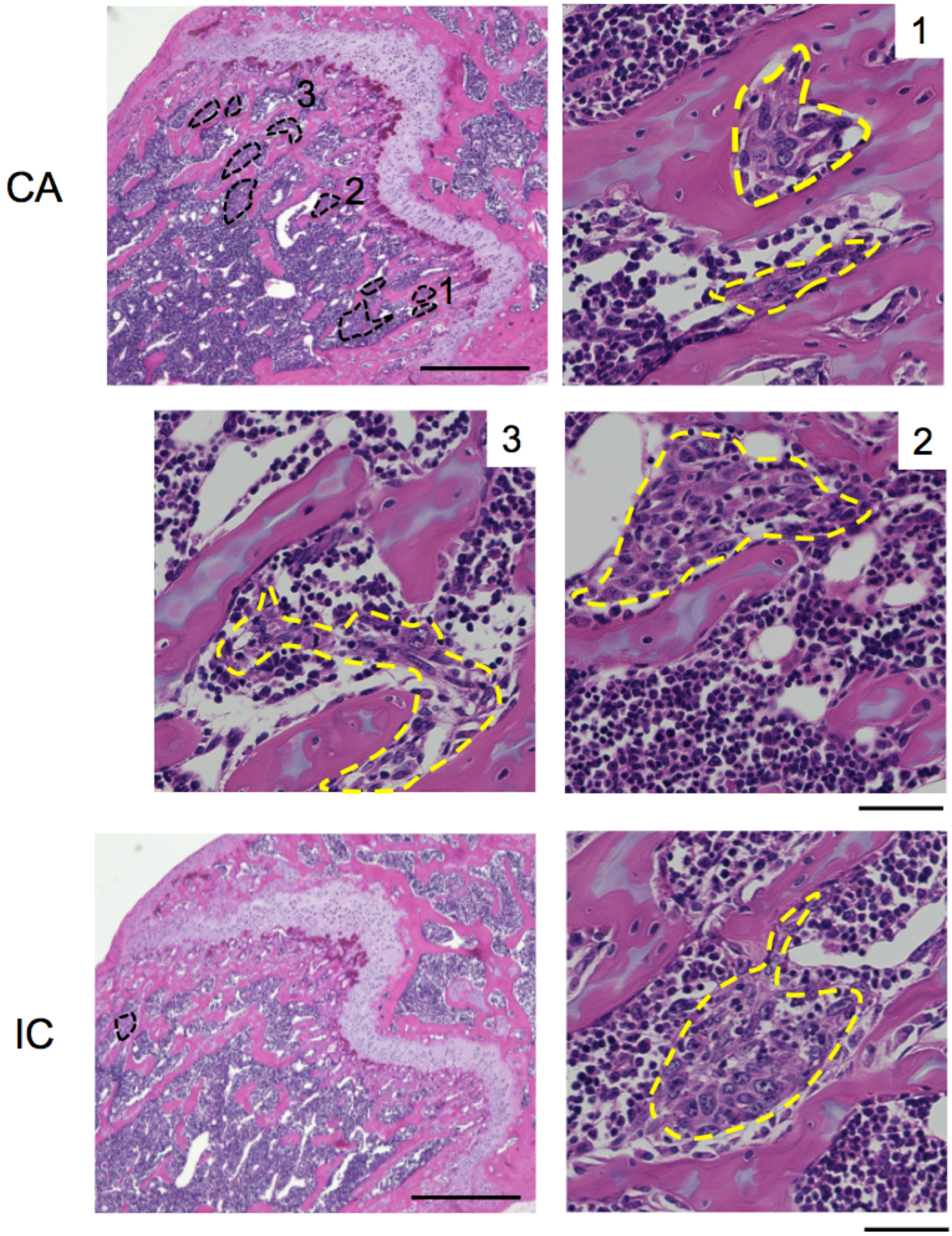

Supplementary Figure 4

Histological analysis of bone metastatic lesions. Representative HE images of bone metastatic lesions in hind limb bones at 7 days after injecting LLC/luc cells via CA or IC. Metastatic lesions were indicated with dashed lines in low-magnification images.

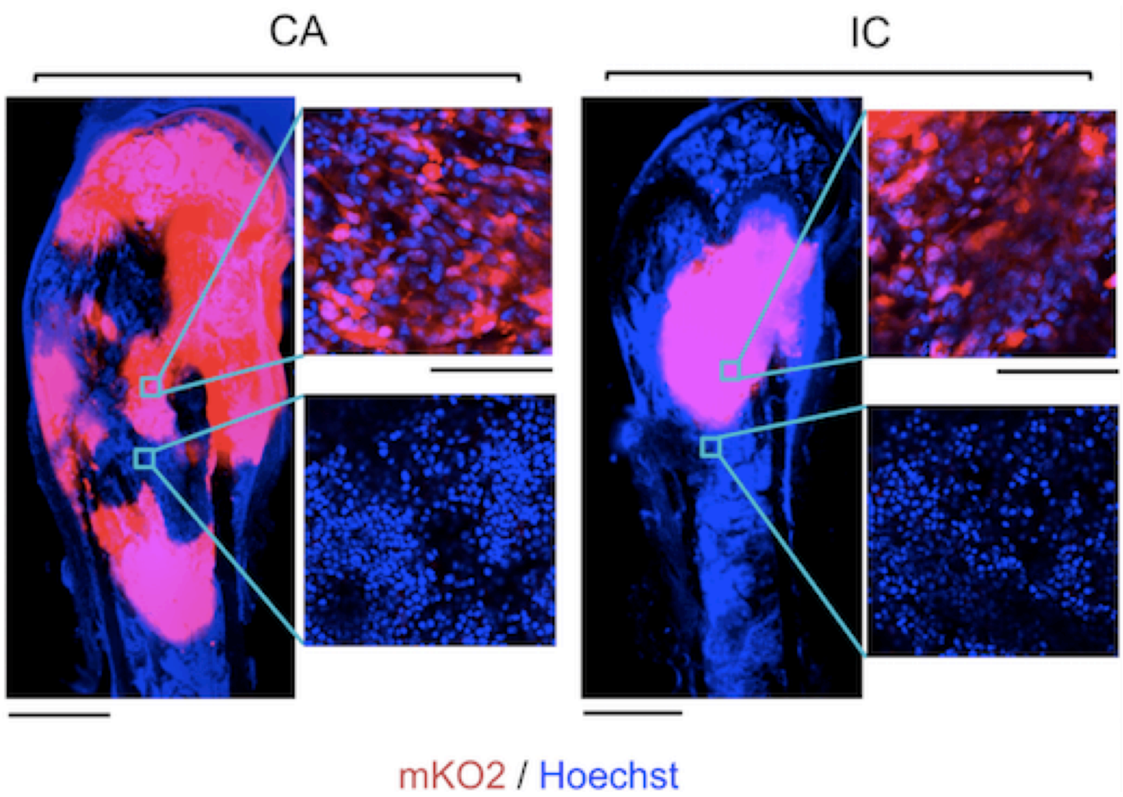

Supplementary Figure 5

Histological analysis of overt bone metastasis. Representative confocal fluorescence images of magnified bone metastatic lesions of Fig.2c. Nuclei were stained as blue with Hoechst. Scale bars are 500 μm and 100 μm in low- and high-magnification images, respectively.

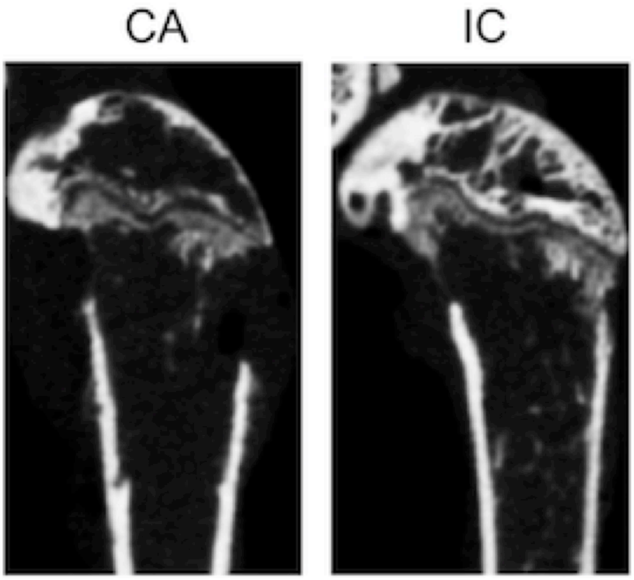

Supplementary Figure 6

X-ray micro CT images of femur with bone metastasis. Representative X-ray micro CT images of sagittal plane of the femur at 14 days after injecting LLC/mKO2-Rluc8.6 cells via CA or IC route.

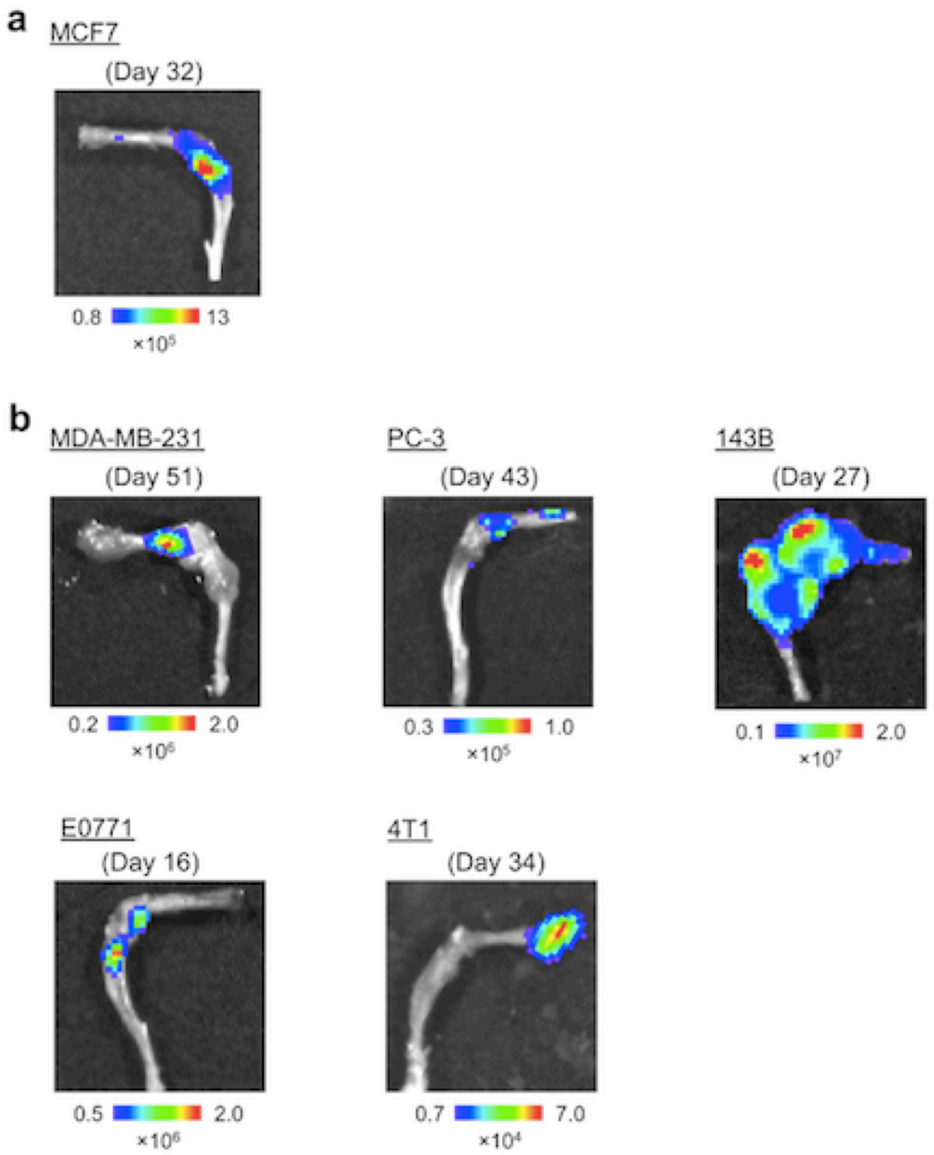

Supplementary Figure 7

***Ex vivo* images of hind limbs with bone metastasis after CA injection.**

(**a**) A representative *ex vivo* image of hind limbs at 32 days after CA injection of MCF7. (**b**) Representative *ex vivo* images of hind limbs at indicated days after CA injection are shown.

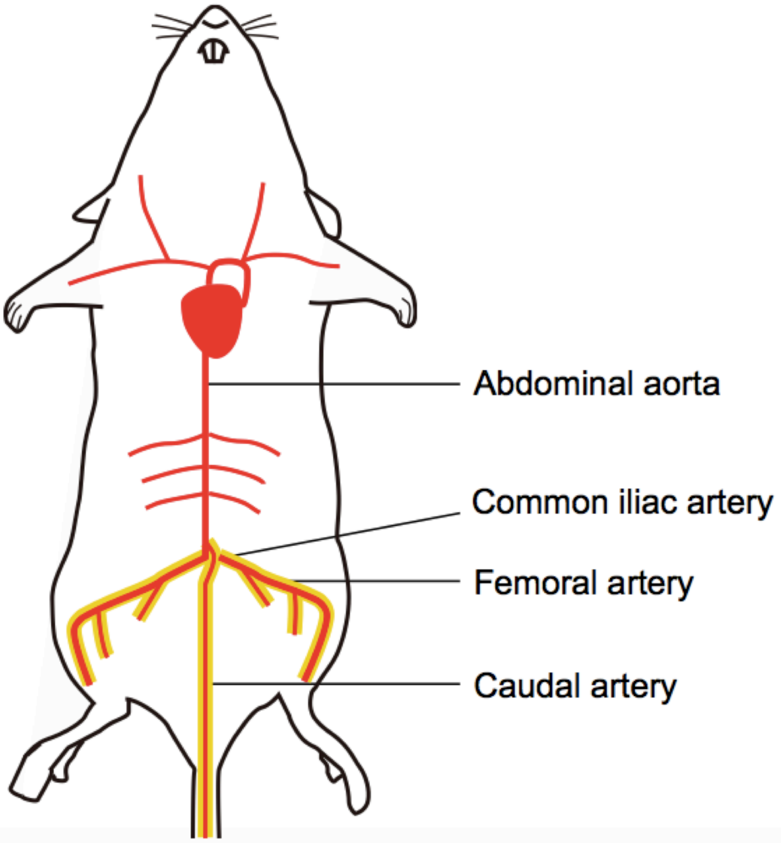

Supplementary Figure 8

A schematic diagram of arterial circulatory system. Representative arteries are shown in red and the ones involved in CA injection are highlighted in yellow.
