# Supplementary material for: A reliable murine model of bone metastasis by injecting cancer cells through caudal arteries
Source: Nat Commun. 2018 Jul 30;9:2981. doi: 10.1038/s41467-018-05366-3 (PMC6065368; doi:10.1038/s41467-018-05366-3)
Supplement: Supplementary file 2 — Description of Additional Supplementary Files [file 41467_2018_5366_MOESM2_ESM.pdf]

Description of Additional Supplementary Files

File Name: Supplementary Movie 1
Description: Mouse being injected with NIR-II nanoparticles via CA

File Name: Supplementary Movie 2
Description: Mouse being injected with NIR-II nanoparticles via IV

File Name: Supplementary Movie 3
Description: CA injection of cancer cells into a mouse under anesthesia
